# Reviews

**Published:** 1871-11

**Authors:** Wm. Abram Love

**Affiliations:** Atlanta, Georgia


### REVIEWS.

BY WM. ABRAM LOVE, M.D., ATLANTA, GEORGIA.

THE AMATEUR MICROSCOPIST, OR VIEWS OF THE MICROSCOPIC WORLD. A Hand-Book of Microscopic Manipulations and Microscopic Objects. By JOHN BROCKLESBY, A.M., Professor of Mathematics and Natural Philosophy in Trinity College, Hartford; Author of "The Elements of Meteorology," etc. Illustrated with Two Hundred and Forty-seven Figures on Wood and Stone. New York: William Wood & Company, No. 27 Great Jones Street. 1871. pp. 144. (From the Publishers.)

It is with pleasure we hail the publication of such works as this, believing that they are calculated, in an eminent degree, to awaken in the *minds of the people*, an interest in the mysterious revelations of the microscope, and to a better appreciation of the beauty and harmony of Nature, which lie buried in its minutiæ, beyond the reach of the unaided eye. The author, in his preface, says:

"I have been led to believe that a popular work on the microscope and its revelations would at once be interesting and useful, and this belief has resulted in the present treatise, which simply exhibits and describes some of the most rare and curious objects of the microscopic world, and the mode of preparing them for observation under the microscope; together with a short account of this instrument." * * *

He further says:

"A knowledge of the wondrous revelations of the microscope cannot but be interesting; yet I trust that the perusal of this little volume may subserve a higher purpose than to while away an idle hour: that it will enkindle in the reader a desire to use this noble instrument, and by its aid to explore for himself the hidden realms of Nature. A few years ago the microscope was simply regarded as a costly toy, but now its value is appreciated in almost every department of physical science. The information it affords the physician in reference to the tissues of the human body, the nature of disease, and the constitution of the blood, is beyond all price."

No one can appreciate the truth and force of this last sentence of the author so well as the physician who, having followed with the microscope such men as Ehrenberg, Prichard, Carpenter, Quekett, or the world-renowned Beale, in tracing Nature down to her most minute organisms, learned her physiological forms and laws and pathological deviations, and then learned thus to trace obscure and hidden diseases to their origin and seat.

What the telescope, aided by mathematics, has accomplished for the science of astronomy, the microscope, aided by chemistry, is fast accomplishing for the science of medicine; and the time is rapidly approaching when the physician who fails to heed these revelations will find himself far in the background when he strives to arrive at a correct understanding of the true pathology and treatment of masked and obscure maladies.

The author of this little work for amateurs opens with a description of the instrument and mode of mounting objects; runs rapidly through infusorial animalcules and protophytes, then takes up fossils and aquatic animals—while these objects are well illustrated by wood cuts—the plates, representing "the structure of wood and herbs," "crystalizations" and "parts of insects and miscellaneous objects," are well and faithfully executed. The mechanical execution of the work speaks every way well for the publishers. To the amateur and beginner, we would commend it as a work giving much information, in a convenient and readily accessible form.

Transactions of the Medical Association of the State of Alabamb. Annual Session of 1871, held in Mobile, March 21st, 22d, 23d. pp. 356.

To the present worthy President of this Association, Dr. T. C. Osborn, of Greensboro, Alabama, we are indebted for a copy of these "Transactions."

From the character of the scientific papers presented in this volume, Alabama has reason to feel proud of her Medical Association. We had the pleasure of attending the meeting immediately preceding this, and were speedily convinced of the fact that this body was composed of *working men*—men from whose labors in the field of Science the profession might hope to reap in the harvest time. The papers then presented were fraught with interest to every Southern practitioner, and those presented on kindred subjects in the pages before us are not less interesting.

Passing over the subject of "Medical Education," which seems to have more than rippled the "waves of Science," that were wont to flow so smoothly on in this channel, we find the Annual Address of the President, Dr. Ross, of Mobile, replete with interest and wholesome advice to his fellows, admonishing them to combine to work together, making due allowance, in a spirit of charity, for human frailties, and labor upon the certainty that "time will make all things right."

The Annual Orator, Dr. Wm. H. Anderson, draws before his audience a beautifully-painted panorama of the progress of our Science in these later years. He throws his lights and shades with a master hand, acquits himself in a manner becoming the man, and shows that it is a pleasing task—the pursuit of this Science of ours. He burns no incense to "strange gods."

Following this, come the "Reports on the Diseases and Surgery of the Different Counties in the State." This is an interesting feature in the Alabama Association. In these papers is presented much valuable information touching the topography, climate, and the character of the epidemics and endemics of the different sections in the State, and the report of many interesting cases, medical and surgical, occurring in the practice of the different reporters or their professional friends.

Not the least replete with interest, as unique, among these papers, is one on the "Chemical Explanation of Malaria," by Joseph A. Groves, M.D., of Liberty Hill. To those who have given attention to this subject, viewing it from any neighboring stand-point, this paper will present a field open to further and still more interesting research. Were we near enough, we would touch the Doctor's elbow and whisper into his ear, "*ozone*" and "*antozone*," with a word of good cheer, if it would encourage him in his labors. To the Southern physician, this subject of *malaria* is one of the deepest interest, and we hail with pleasure any glimmer of a ray of light that may give token of a hope that the obscuring veil may be penetrated.

The "Topography, Meteorology and Diseases of Hale County," by T. C. Osborn, M.D., of Greensboro, is a model paper of its kind. Accompanied with an outline map of the county as it is, and a topographical description, together with tabulated mortuary, statistical and meteorological reports for the year, a solid foundation is here laid and material stored for the future medical historian of the State. We regret that we have not the time and space to give in full that part of his paper treating of "*malaria*," and can only express the hope that we may be able to do so in some future number of the JOURNAL. Dr. Osborn has presented to the profession, heretofore, several papers on this subject, not wanting either in interest or originality, and it is hoped that, in the same field of research, his labors will be extended.

Dr. Jerome Cochran, the indefatigable Secretary of the Association, presents an elaborate paper on the "Endemic and Epidemic Diseases of Mobile," in which he gives some wholesome advice to the "city fathers" that might, with benefit, be heeded by the "fathers" of some other cities we wot of, if they would!

Many other papers in these "Transactions" we would gladly refer to in an extended manner here, but time and space forbid; they may command our attention anon.

